# Clinical landscape of cancer metastases

**DOI:** 10.1002/cam4.1697

**Published:** 2018-10-16

**Authors:** Matias Riihimäki, Hauke Thomsen, Kristina Sundquist, Jan Sundquist, Kari Hemminki

**Affiliations:** ^1^ Division of Molecular Genetic Epidemiology German Cancer Research Centre (DKFZ) Heidelberg Germany; ^2^ Center for Primary Health Care Research Lund University Malmö Sweden; ^3^ Department of Family Medicine and Community Health Department of Population Health Science and Policy Icahn School of Medicine at Mount Sinai New York City New York; ^4^ Department of Functional Pathology Center for Community‐based Healthcare Research and Education (CoHRE) School of Medicine Shimane University Matsue Japan

**Keywords:** cancer, database, epidemiology, metastasis, nationwide

## Abstract

Population‐based data on metastatic patterns are lacking because cancer registries seldom record metastases. This study uses a novel population‐based approach to identify metastases and describes metastatic pathways from 14 common primary cancers to 12 specific metastatic sites. A total of 179 581 patients with metastatic cancer were identified from the Swedish Cancer Registry and metastatic sites were identified using the Cause of Death Register and the National Patient Register. Patterns of metastatic spread were described across age and sex. In men, colorectal cancer was the main source of lung, peritoneal, and liver metastases. Lung cancer was the main origin of pleural and nervous system metastases. Prostate cancer dominated bone metastases but had minor contribution to other metastatic sites. Among women, breast cancer was the dominant origin of most metastatic sites, with the exception of peritoneum which was ruled by metastases from the ovary. As other exceptions, for nervous system metastases, lung cancer was the origin of metastases somewhat more frequently than breast cancer and for liver metastases, colorectal cancer was the main origin instead of breast cancer. The present achievement was to implement the first nationwide description of clinical landscape of cancer metastases, with an aim to serve as a reliable source for clinicians and researchers.

## 1 INTRODUCTION

Recent progress in cell and molecular biology on cancer metastasis has revealed important components of the process and their bewildering complexity. The results have refined the old seed and soil and anatomical/mechanical hypotheses with data on tissue invasion, stem cell characteristics, cell plasticity, epithelial‐mesenchymal transition, immunoediting and dormancy with interacting genes and cell types.^1^, ^2^, ^3^, ^4^, ^5^, ^6^ Genomewide sequencing has been able to describe clonal evolution of metastases in many cancers.^7^, ^8^, ^9^ The data suggest that mutational patterns differ between primary tumors and metastases and between metastases which may have implications to response to therapy and selective seeding of metastases.^4^, ^6^, ^8^, ^10^ However, major questions remain, for examples, on the mechanisms guiding the metastatic patterns between primary cancers and selective locations of metastases.

The increasing molecular understanding of the metastatic process has not helped to boost epidemiology of metastases. The available data on the distribution of metastases originate from hospital or autopsy series or insurance claims.^9^, ^11^, ^12^, ^13^, ^14^ All these data sources are based on certain type of selection and autopsy rates have decreased in many countries to the level that case numbers are limiting. The scarcity of population‐based data compared to primary cancers is due to the fact that most cancer registries do not collect information on metastases beyond the tumor, node, metastasis (TNM) classification which dichotomously gives the presence or absence of metastases without locations at the time of diagnosis. As alternative sources on data on metastases, we have used death certificates but there may be concerns that these are issued to describe the causes of death and the focus may be on life‐threatening types of metastases.^15^ We and others have also used hospital discharge records which at least in Sweden and Denmark are useful and accurate but have the disadvantage that the time of diagnosis of cancer or of metastases may not be available, complicating survival analysis.^15^, ^16^, ^17^, ^18^, ^19^ Using Swedish hospital inpatient data combined with data from death certificates, we show here the spectrum of metastases from all common cancers. To our knowledge, this is the first population‐based study of its kind providing information on 179 581 site‐specific extranodal metastases disseminating from common cancers.

## 2 MATERIAL AND METHODS

### 2.1 Dataset

The Swedish Family‐Cancer Database (FCD) includes cancer data from the Swedish Cancer Registry, and information of death causes from the Cause of Death Register.^20^ The primary cancers are coded by the International classification of diseases’ (ICD) 7th revision in the Cancer Registry. SNOMED histological codes and TNM staging is included for cancer patients diagnosed after 2002. The National Patient Register includes data from all hospitalizations in Sweden, with nationwide coverage since 1987.^21^ Two sources were used for identifying metastatic involvement. In the National Patient Register, metastases can either be listed as the main diagnosis or accompanying diagnoses during the hospitalization. The National Patient Register includes up to 21 supporting diagnoses. In addition to diagnoses, the National Patient Register also includes any procedures performed during the hospitalization. Reporting to the National Patient Register is obligatory in both public and private healthcare centers.^21^ Alternate sources of metastatic data were causes of death, which were identified from the national Cause of Death Register. Here, the underlying cause of death was listed together with up to 10 accompanying causes of death. Between 1987 and 1996, coding in the National Patient Register and death certificates was done according to ICD version 9 coding. Since 1996, ICD‐10 has been used. ICD codes used for identifying metastases are displayed in Table 1. Coding is easily translated between ICD‐9 and ICD‐10. The available versions of the National Patient Register and FCD include all new cancers and hospitalizations until the end of 2012. Therefore, this analysis was restricted from year 1987 to 2012. Patients with unknown primary and multiple primary sites were excluded. Similar analyses already are available for cancer of unknown primary.^22^

**Table 1:** Number of patients and median age at diagnosis (in years) of primary cancer and site of extranodal metastasis. Because patients may have several metastases, the sum of metastases is larger than that of patients

Primary cancer	Male			Female			All		
	N	Col %	Median age at diagnosis	N	Col %	Median age at diagnosis	N	Col %	Median age at diagnosis
Upper aerodigestive	1718	2	63.8	791	1	65.6	2509	1	64.3
Esophagus	1770	2	66.1	489	1	69.6	2259	1	66.6
Stomach	4088	4	69.7	2484	3	71.2	6572	4	70.3
Colorectum	16 962	18	69.7	14 429	17	71.3	31 391	17	70.3
Liver	2524	3	69.4	3129	4	71.8	5653	3	70.7
Pancreas	4240	5	68.3	4121	5	70.3	8361	5	69.3
Lung	13 951	15	68.0	11 276	13	66.5	25 227	14	67.3
Breast	0			25 594	29	60.0	25 594	14	60.0
Other female genital	0			5867	7	67.3	5867	3	67.3
Ovary	0			6257	7	64.3	6257	3	64.3
Prostate	28 936	31	72.8	0			28 936	16	72.8
Kidney	4365	5	66.1	3000	3	68.9	7365	4	67.3
Bladder	3284	4	71.2	1157	1	72.9	4441	2	71.5
Melanoma	3015	3	63.3	1908	2	61.5	4923	3	62.6
Other^a^	7860	8	64.4	6366	7	67.8	14 226	8	66.1
All above	92 713	100	69.8	86 868	100	66.3	179 581	100	68.3

Location of metastasis	ICD‐9 code	ICD‐10 code	Male		Female		All	
			N	% of total	N	% of total	N	% of total
Lung	197.0	C78.0	19 191	15	22 931	16	42 122	15
Pleura	197.2	C78.2	3256	2	6729	5	9985	4
Other respiratory	197.1/3	C78.1/3	1786	1	1839	1	3625	1
Peritoneum	197.6	C78.6	5882	4	12 156	9	18 038	7
Liver	197.7	C78.7	32 160	24	34 346	24	66 506	24
Other G‐I	197.4‐5/8	C78.4‐5/8	4481	3	5591	4	10 072	4
Urinary system	198.0/1	C79.0/1	1746	1	1355	1	3101	1
Skin	1982	C79.2	1851	1	4037	3	5888	2
Nervous system	198.3/4	C79.3/4	11 890	9	13 965	10	25 855	9
Bone	198.5	C79.5	39 126	30	24 218	17	63 344	23
Ovary	198.6	C79.6	0	0	1395	1	1395	1
Adrenal gland	198.7	C79.7	2202	2	1769	1	3971	1
Other	198.8	C79.8	8335	6	11 099	8	19 434	7
Sum of all metastases			131 906	100	141 430	100	273 336	100

a Other ICD‐7 codes 140‐209 except code 199 (cancer of unknown primary). Col%, percent distribution of patients. Other G‐I, other gastro‐intestinal location.

**Figure 1:**
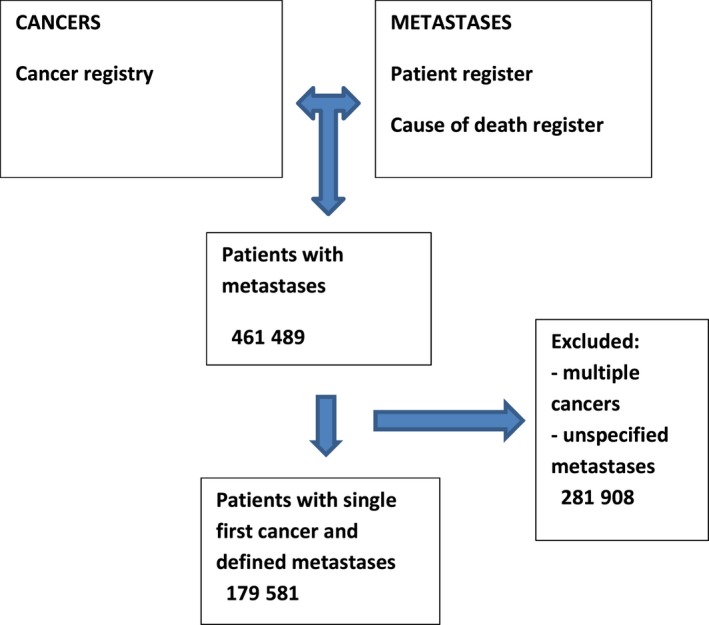
A scheme for data linkages used to identify the patient population with defined metastases for this study

Linkage of data is illustrated in Figure 1. Patients with cancer were identified from the Cancer Registry and these were linked with the National Patient Register and the Cause of Death Register to identify unique patients with extranodal metastases. A total of 461 489 such patients were identified but 281 908 were excluded for reasons such as they had more than one cancer, or the diagnosis was cancer of unknown primary. Other reasons for exclusion were unspecified or ill‐defined metastases.

### 2.2 Statistical analysis

We created a subset of the National Patient Register and FCD with all patients with a recorded hospitalization, or death, due to metastatic cancer between 1987 and 2012. First, the primary cancer sites followed by metastatic sites were investigated for both sexes. Patients may have been hospitalized multiple times due to the same metastases, and also with metastases to multiple locations. Separate analyses were performed taking into account the age at diagnosis of the primary cancer (three groups: <60 years, 60‐70 years, and ≥70 years).

As a quality control test, we used the TNM classification, initiated in the Cancer Registry in 2002, to identify metastatic stage IV colorectal cancer patients, and search for information on their metastatic locations through the Cause of Death Register and the National Patient Register. We assume that the data in the Cancer Registry are correct and can check to what extent the patients can be correctly identified through the sources that we used to identify metastases.

All calculations were performed using SAS software, version 9.3 (SAS Institute Inc., Cary, NC).

## 3 RESULTS

A total of 1.25 million patients who were diagnosed between 1987 and 2012 were identified from the Cancer Registry. We excluded patients with more than one primary cancer diagnosis (N = 131 565) and those with an unknown primary site (N = 34 100). Furthermore, we excluded patients with unspecified metastases (N = 113 890) or metastases to “ill‐defined” sites (N = 2353). Of remaining cancer patients, 179 581 had records of extranodal metastasis: 116 424 had one, 41 545 had two, and 21 612 had three or more known metastases. The overall median age at cancer diagnosis was 68.3 years (Table 1). The most common primary cancers were prostate (31%), colorectum (18%), and lung (15%) cancers for men. The most frequent primary sites for women were breast (29%), colorectum (17%), and lung (13%). In men, the skeleton was the target of most metastases, followed by liver and lung, whereas in women, the liver was the main target, followed by bone and lung.

Table 2 summarizes the primary cancer sites in men with metastatic cancer (N = 92 713). The first line gives the total distribution of metastases, lead by bone (42%) and followed by liver (35%) and lung (21%). Note that the sum exceeds 100% because many patients had multiple metastases which were scored to multiple sites. Lung metastases were most common organ sites for upper aerodigestive tract, esophageal, and kidney cancers. The liver was the most common target for stomach, colorectal, liver, and pancreatic cancers. The nervous system was preferred location of metastases from lung cancer and melanoma, and the bone was targeted by prostate and bladder cancers. As many as 89% of prostate cancer patients showed bone metastases while the next common site of the liver was the target in only 10% of patients. For other cancers, the distribution of metastatic locations was more even; for example for lung cancer, nervous system (39%) and bone (34%), and for melanoma nervous system (49%) and lung (41%) were rather even locations.

**Table 2:** Location of primary cancer in 92 713 male cancer patients, depending on the site of metastasis

Location of primary cancer	Location of metastasis																								
	Any metastasis	Lung		Pleura		Other respiratory		Peritoneum		Liver		Other G‐I		Urinary		Skin		Nervous system		Bone		Adrenal gland		Other	
	N	N	%	N	%	N	%	N	%	N	%	N	%	N	%	N	%	N	%	N	%	N	%	N	%
Total	92 713	19 191	21	3256	4	1786	2	5882	6	32 160	35	4481	5	1746	2	1851	2	11 890	13	39 126	42	2202	2	8335	9
UAT	1718	783	46	65	4	72	4	30	2	357	21	51	3	16	1	112	7	209	12	402	23	28	2	370	22
Esophagus	1770	607	34	78	4	110	6	95	5	916	52	99	6	16	1	35	2	163	9	346	20	42	2	152	9
Stomach	4088	573	14	145	4	56	1	938	23	2287	56	430	11	20	0	63	2	167	4	404	10	63	2	363	9
Colorectum	16 962	5351	32	261	2	133	1	2236	13	12 189	72	1589	9	374	2	250	1	1019	6	1479	9	233	1	1304	8
Liver	2524	550	22	67	3	12	0	389	15	1522	60	161	6	21	1	29	1	70	3	352	14	68	3	188	7
Pancreas	4240	657	15	138	3	16	0	610	14	3356	79	309	7	36	1	24	1	54	1	255	6	55	1	295	7
Lung	13 951	1493	11	1450	10	776	6	202	1	3376	24	350	3	256	2	280	2	5423	39	4720	34	1065	8	1260	9
Prostate	28 936	1983	7	265	1	100	0	287	1	2751	10	413	1	364	1	122	0	1155	4	25 627	89	106	0	2197	8
Kidney	4365	2475	57	230	5	165	4	160	4	900	21	199	5	266	6	97	2	800	18	1600	37	247	6	415	10
Bladder	3284	1011	31	77	2	42	1	191	6	1031	31	163	5	159	5	53	2	266	8	1354	41	55	2	426	13
Melanoma	3015	1245	41	112	4	79	3	130	4	877	29	265	9	56	2	531	18	1469	49	547	18	127	4	466	15
Other	7860	2463	31	368	5	225	3	614	8	2598	33	452	6	162	2	255	3	1095	14	2040	26	113	1	899	11

Note

UAT, upper aerodigestive tract; Other G‐I, other gastro‐intestinal location.

For female metastatic locations, the ranking differed from men because the liver was the most affected site, followed by the bone and the lung. However, for the shared cancers, the ranking of metastatic locations matched the male one. Among female cancers, breast cancer metastasized to bone (55%), liver (36%), and lung (30%). Other female genital cancers showed metastatic growth to lung (36%) and peritoneum (26%). Ovarian cancer spread mainly locally in the peritoneum (62%).

**Figure 2:**
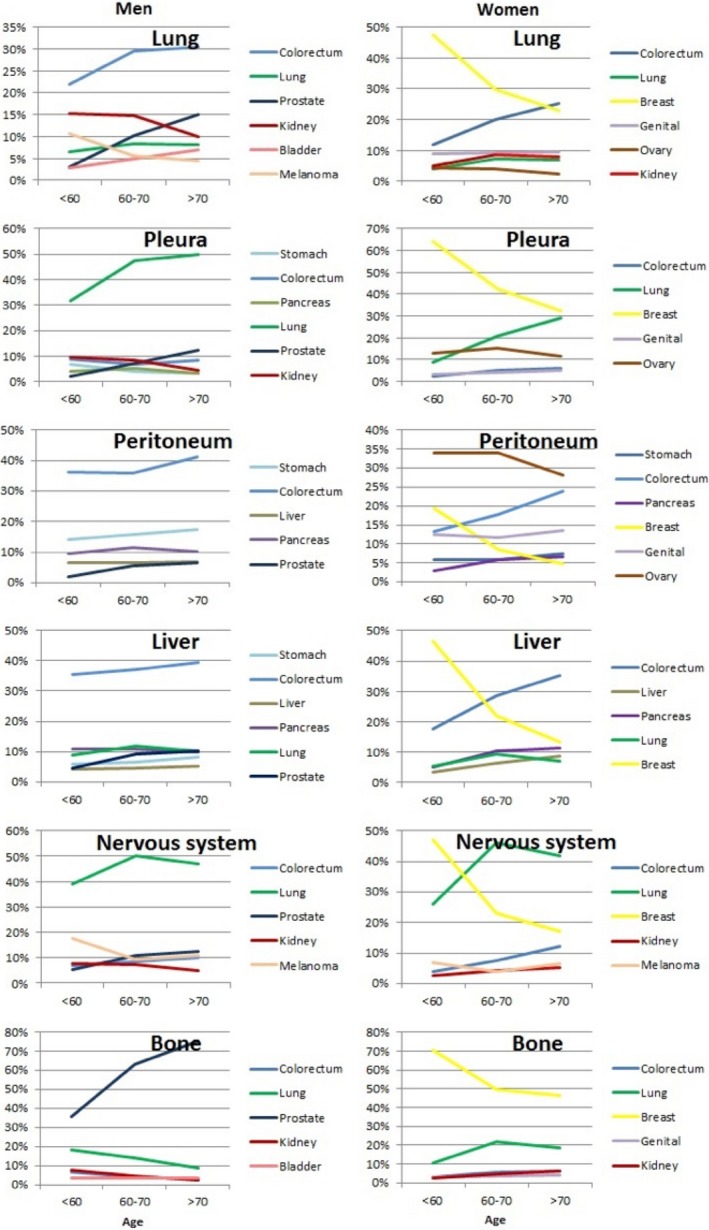
Location of primary cancer depending on the sites of metastasis across three age groups: <60 y, between 60 and 70 y, and 70 y or more. The *y*‐axis shows the percentage of all metastases; “age” refers of age at diagnosis. Note that “other cancers,” not shown, are part of 100%

Figure 2 depicts patterns of metastases as percentage of all metastases depending on the diagnostic age of cancer. Of note, “other primary sites” account for a substantial proportion of metastases in younger men and these were not shown. For example, 20% of lung metastases in men under 60 years were from “other” sites, whereas the proportion was only 9% in men over 70 years. In women, other sites caused 7% of lung metastases in all age groups. In men, colorectal cancer was the main source of lung, peritoneal, and liver metastases with moderately increasing share toward high age. Lung cancer was the main origin of pleural and nervous system metastases which also increased moderately to age 60‐70 years. Prostate cancer dominated bone metastases at all ages but most at high age. Among women, early onset breast cancer was the dominant origin of all metastatic sites, with the exception of peritoneum which was controlled by metastases from the ovary. Lung cancer overtook breast cancer as the main origin of nervous system metastases past age 60 years. Based on Table 3, lung cancer contributed somewhat more nervous system metastases than breast cancer; similarly, for liver metastases, colorectal cancer slightly surpassed breast cancer as source of metastases.

**Table 3:** Location of primary cancer in 86 868 female cancer patients, depending on the site of metastasis

Location of primary cancer	Location of metastasis																										
	Any metastasis	Lung		Pleura		Other Respiratory		Peritoneum		Liver		Other G‐I		Urinary		Skin		Nervous system		Bone		Ovary		Adrenal gland		Other	
	N	N	%	N	%	N	%	N	%	N	%	N	%	N	%	N	%	N	%	N	%	N	%	N	%	N	%
Total	86 868	22 931	26	6729	8	1839	2	12 156	14	34 346	40	5591	6	1355	2	4037	5	13 965	16	24 218	28	1395	2	1769	2	11 099	13
UAT	791	381	48	30	4	28	4	11	1	150	19	25	3	14	2	62	8	109	14	191	24	1	0	9	1	184	23
Esophagus	489	191	39	23	5	36	7	23	5	210	43	30	6	3	1	8	2	43	9	77	16	1	0	13	3	38	8
Stomach	2484	276	11	98	4	25	1	799	32	1097	44	329	13	22	1	50	2	68	3	236	10	161	6	22	1	267	11
Colorectum	14 429	4393	30	281	2	124	1	2260	16	9507	66	1469	10	214	1	259	2	979	7	1149	8	424	3	145	1	1445	10
Liver	3129	505	16	77	2	18	1	523	17	2216	71	271	9	15	0	50	2	53	2	211	7	34	1	33	1	224	7
Pancreas	4121	727	18	139	3	17	0	638	15	3097	75	331	8	31	1	27	1	62	2	211	5	29	1	54	1	321	8
Lung	11 276	1387	12	1223	11	605	5	153	1	2466	22	276	2	180	2	265	2	4999	44	3827	34	26	0	852	8	1068	9
Breast	25 594	7703	30	3271	13	544	2	1320	5	9133	36	864	3	191	1	2250	9	4583	18	14 026	55	273	1	227	1	2863	11
Other genital	5867	2120	36	273	5	104	2	1538	26	1219	21	470	8	195	3	197	3	477	8	871	15	106	2	73	1	1620	28
Ovary	6257	814	13	874	14	69	1	3853	62	1282	20	818	13	74	1	139	2	315	5	234	4	221	4	37	1	1482	24
Kidney	3000	1584	53	98	3	88	3	122	4	711	24	157	5	208	7	71	2	532	18	1045	35	15	1	130	4	334	11
Bladder	1157	361	31	30	3	25	2	97	8	304	26	59	5	57	5	28	2	73	6	435	38	3	0	18	2	175	15
Melanoma	1908	746	39	60	3	40	2	89	5	516	27	122	6	30	2	427	22	848	44	300	16	15	1	64	3	292	15
Other	6366	1743	27	252	4	116	2	730	11	2438	38	370	6	121	2	204	3	824	13	1405	22	86	1	92	1	786	12

Note

UAT, upper aerodigestive tract; Other G‐I, other gastro‐intestinal location.

In order to assess the reliability of our data, we analyzed through the TNM classification data a sample of 8131 stage IV colorectal cancer patients diagnosed in 2002 through 2012. Of these, 83% had records of site‐specific metastases, and over 5% had metastases at ill‐defined, lymphatic, or unspecified sites. As 5% of these had had not died, we conclude that the underreporting of TNM defined metastases in the combined registries was around 7%.

## 4 DISCUSSION

The present study provides an estimate of metastatic pathways from primary cancers to main metastatic sites. The advances are the nationwide, unselected, and recent patient population of 179 581 individuals. To our knowledge, no such data have been published before. The limitations of the study are that the diagnoses were clinical, not pathological, and that a proportion of metastatic patients were probably not reported. In the quality control analysis, we estimated underreporting of metastatic colorectal cancers at 7%. It is important to note that we describe metastatic pathways from primary cancer rather than attempt to describe the risk of metastases from each specific primary cancer, because such figures would be underestimated.

Our findings of preferred metastatic sites of primary cancers are largely in line with clinical knowledge and the reported autopsy data but they have never been presented at a national level.^12^, ^13^, ^14^ Because of sex‐specific cancers and the dominance of prostate cancer in men with preferred bone metastases, the overall male raking of metastatic sites was bone, liver, and lung compared to liver, bone, and lung in women. The contribution on the main primary sites to metastasis at various sites is shown in the nutshell in Figure 2. In men, colorectal cancer was the main source of lung, peritoneal, and liver metastases. Lung cancer was the main origin of pleural and nervous system metastases. Prostate cancer dominated bone metastases and had a moderate contribution lung cancer but had minor contribution to other metastatic sites. Among women, breast cancer was the dominant origin of all metastatic sites, with the exception of peritoneum which was ruled by metastases from the ovary. Figure 2 does not reveal two other exceptions that can be found in Table 3: for nervous system metastases, lung cancer was the origin of metastases somewhat more frequently than breast cancer, and for liver metastases, colorectal cancer was the main origin instead of breast cancer. Breast cancer is a relatively early onset cancer and Figure 2 showed that its dominance at many metastatic locations was weakened toward higher age.

The present results agree with earlier autopsy studies showing that gastro‐intestinal cancers primarily metastasize to the liver, and that prostate, breast, lung, and kidney cancer are the main sources of bone metastases.^12^, ^13^, ^14^ In the present study, most liver metastases arose from colorectal cancer, as was also reported by Hess et al^13^; however, earlier studies have suggested breast and lung cancers as the main source of liver metastases.^12^, ^14^ Breast and colorectal cancers have been the dominant sources of lung metastases in the literature, although Hess et al reported a substantially higher proportion of lung metastases from kidney cancer (28% of all lung metastases) compared with the present results (13% for men, 7% for women). There is also agreement that lung cancer and breast cancers are dominant sources of nervous system metastases. However, we show here that for melanoma, the nervous system was the most common metastatic site found in close to a half of all patients.

The accuracy of the data is vital for this study. Some 98% of cancer cases in the Cancer Registry have been cytologically verified and the coverage has been estimated at over 90%.^23^ Causes of death have been registered in Sweden since the 18th century.^24^ The recent completeness of the Causes of Death Register has been estimated to be higher than 99% but inaccuracies may exist in rendered diagnoses.^18^ Previous Swedish studies showed that cancer deaths showed the highest agreement with hospital diagnoses.^25^, ^26^ A contributing factor to the accuracy of death certificates on cancer patients in Sweden is that 85% of patients die in hospitals and for more than 90% of cancer deaths the related hospital journals have been the base for issuing the death certificate.^27^, ^28^ In the presented data, 34 100 of patients with records of metastatic involvement had metastases to “unspecific” sites. Although this may raise some concerns, this issue has been addressed in a previous study concerning metastatic lung cancer.^15^ There was no association between factors such as age, sex, survival, socio‐economic index, geographic location, or histological type on the occurrence of metastases to “unspecific” sites. Therefore, we are confident that metastases to “unspecific” sites should be considered random in nature.

The “anatomical/mechanical” hypothesis and the “seed and soil” hypotheses show merit in practice. The anatomical/mechanical hypothesis explains the location of lymphatic spread, for example, to axillary lymph nodes from breast or lung cancer. Similarly, due to the portal venous system, many gastro‐intestinal organs metastasize to the liver. Intra‐abdominal cancers from the colorectum, the ovaries, or the stomach, often metastasize within the abdominal cavity and lung cancer within the thorax. As all blood passes the lungs, cancer cells from any organ would be expected to seed to the lungs. Anatomical circumstances can also explain why some metastases appeal concurrently to each other, for example, ovary and peritoneum/gastro‐intestinal, but not other pairs, such as adrenal gland and nervous system. Some cancer may prefer to target organs with a similar milieu. Depending on the “soil” (target organ), different “seeds” may thrive. For example, we recently showed that different histological subtypes in lung cancer displayed significant difference in metastatic patterns, irrespective of age and sex.^15^ Adenocarcinomas frequently metastasized to bone. Small cell lung cancer metastasized to the liver and nervous system, containing neuroendocrine cells. Some authors emphasize the role of disseminating tumor cells in acquiring genetic and epigenetic variation in distant locations that enable metastatic expansion; they referred to this process as “metastatic speciation.”^29^

Recently, the seed and soil hypothesis have evolved into increasing complexity, as factors mediating metastasis spread were investigated at the molecular level, increasing the importance of understanding target organ microenvironment as an important factor in metastasis.^4^, ^5^, ^10^, ^30^, ^31^, ^32^ Expression of several genes may generally enhance a primary tumors metastatic potential or even facilitate the emergence of metastatic traits.^10^ Overexpression of growth factor receptors, cell adhesion molecules, and chemoattractants mediating cancer cell homing in different cancers may furthermore promote spread generally or to specific organs that display a suitable microenvironment.^4^, ^5^, ^10^, ^30^, ^31^, ^32^ Tumor cells expressing, for example, fibroblast growth factor receptor 1 or parathyroid hormone‐related peptide have an advantage in metastasizing to bone, because growth is thus stimulated in that microenvironment.^33^, ^34^ Fibroblast growth factors are often produced by breast cancer and prostate cancer, the latter which also produces prostate‐specific antigen, capable of activating growth factors in bone tissue. Adrenal glands have a high expression of the chemokine CCL20, whereas lung cancer cells frequently express its ligand CCR6, possibly explaining the frequency of adrenal metastases from lung cancer.^32^

In conclusion, there are significant differences in metastatic pathways of main cancer and these depend also on sex and age at diagnosis. To our knowledge, this is the first nationwide description of clinical landscape of cancer metastases. It should serve as a reliable source for clinicians and fellow researchers, spawning numerous ideas for further research on mechanisms behind cancer metastasis.

## CONFLICT OF INTEREST

The authors declare no conflict of interest.
